# Cognitive theory development as we know it: specificity, explanatory power, and the brain

**DOI:** 10.3389/fpsyg.2013.00056

**Published:** 2013-02-19

**Authors:** Davide Crepaldi, Simona Amenta

**Affiliations:** MoMo Lab, Department of Psychology, University of Milano-BicoccaMilan, Italy

In an effort to define more precisely what we currently know about early steps in the visual identification of complex words, we recently published a review of morphological effects in lexical decision, unmasked priming and masked priming studies (Amenta and Crepaldi, 2012). The review aims at identifying a set of well-established experimental effects that any theory in the field should be able to explain, so as to allow for a more rigorous adjudication process to take place and for the field to progress incrementally toward more and more explanatory power (Grainger and Jacobs, 1996; Coltheart et al., 2001). We called this set of experimental effects *“the target list”* (Amenta and Crepaldi, 2012, p. 9). Shortly afterwards, Koester (2012) published a commentary that highlights a set of open issues concerning our paper, which we try to address here. The questions raised by Koester (2012) are all well motivated, and their answer strongly influence how the target list is going to be used in future research; for this reason, it is important to address Koester's questions in a timely manner and, in doing so, to specify more clearly why we believe that the target list is important for the field, and how it should be used. Importantly, although we strongly advocated in our original paper for cognitive theories to become computational models, Koester's points apply more generally to any kind of cognitive theory, either computational or descriptive; our replies will thus try to stand at that general level, which stresses the generality and importance of the issues highlighted by Koester (2012).

The first issue raised by Koester (2012) speaks as follows in his own words:

“Amenta and Crepaldi's review points toward relevant linguistic … and psycholinguistics variables … and their relations regarding visual word identification. …. The authors suggest that these findings provide a basis for the evaluation of competing theories and, in doing so, to contribute to future theory development; in their own words, to construct an “all-inclusive model of visual identification of complex words”. In light of the specificity of the insights, these broad suggestions leave the reader with the impression of a gap between insights and suggestions.” (p. 1, 2nd column, 2nd paragraph)

Koester (2012) seems to question that focusing on very specific experimental effects might drive to enlarge the generality of our theories. We suspect that the exact definition of “generality” is the key point here. If a theory is general when it surpasses the boundaries of the field where it was developed (e.g., it is possibly insightful for spoken word identification while it was developed to explain reading data), then Koester is right that focusing on small-scale, specific effects would not help. But if generality is conceived as explanatory power, i.e., a theory is more general than another when it explains more data, then assessing theories on how many relevant experimental effects they are able to explain clearly encourages the development of general models. We acknowledge that it would be desirable to have general theories in the sense endorsed by the former approach. However, “cross-field” generality normally comes at some cost in terms of model under-specification, and current morphological theories lack in details under so many points of view that this is probably a cost we cannot afford at the moment. Theories are useful in the first place because they generate *testable* predictions; the less they are specified, the less likely they will be to generate predictions of this kind.

The second point highlighted by Koester (2012) concerns the role of some variables that are well-established factors in reading, which we left unaddressed in our review

“such as surface frequency, word length, word class, abstractness, or cues to morpheme boundaries.” (p. 1, 3rd column, 1st paragraph)

Of course, Koester is right that these variables are very relevant in the existing literature on visual word identification; however, it is difficult to envisage a specific role for them in theories *that focus on morphology*. Apart from cues to morphemic boundaries—whose effect, however, has never been demonstrated unambiguously, i.e., we do not know about any study where these variables were manipulated *independently* of any other—, these factors are not morphological in nature, and so whether or not any model of visual complex word identification will be able to account for them depends on aspects of the theory that have nothing to do with morphemes. Possibly, surface frequency might be relevant for the morphological aspects of a theory of visual word identification by virtue of its relationship with stem frequency. Indeed, stem and surface frequency were shown to interact in complex word processing (e.g., Baayen et al., 2007). This issue was covered in Amenta and Crepaldi (2012, p. 2).

A third big issue raised by Koester (2012) concerns

“how the neural evidence is to be incorporated into a strictly cognitive model.” (p. 1, 3rd column, 2nd paragraph)

There are two levels, we believe, at which this issue needs to be addressed. In terms of assessing the explanatory adequacy of cognitive theories, i.e., which experimental data any model should be tested against, there seems to be little role for brain data (e.g., fMRI and ERP). Of course, cognitive neuropsychology has indeed proven decisive to inform the structure of cognitive models (and reading models in particular), and often it has provided evidence for theoretical claims in a way that is unrivalled by other disciplines for elegance and simplicity (e.g., Coltheart, 1982; Coslett and Saffran, 1989; Luzzatti et al., 2001). However, this evidence was always behavioral in nature—essentially, response time, and accuracy—, because this is what maps onto the predictions that purely cognitive models can make. In fact, existing cognitive theories of how we identify printed complex words make no explicit statement on the brain structures that underlie the system and on how these structures work (e.g., Taft and Ardasinski, 2006; Gonnerman et al., 2007; Crepaldi et al., 2010; Baayen et al., 2011; Grainger and Ziegler, 2011). Thus, they make no predictions on brain data. This is true more in general for all existing computational models of reading (e.g., Coltheart et al., 2001; Norris, 2006; Davis, 2010): they have no way to model neural responses such as, e.g., ERPs or BOLD signal, and consequently they should not be evaluated on these grounds.

Here the second, more general level at which this issue should be addressed comes about: why is this the case? There seems to be no principled reason behind this fact. Indeed, one would just need some function to link mental computations (of whatever kind) to the activity of brain units (of whatever size, from single neurons to cortical areas) in order to produce quantitative predictions about neural responses on the basis of some kind of cognitive model. The problem is exactly that this link function has been proven extremely difficult to find. Typically, this was related to the idea that the brain uses distributed representations, i.e., even simple concepts/mental objects such as individual words or individual letters are represented through a pattern of activation over an indefinite number of brain units, i.e., neurons or small clusters of neurons (e.g., McClelland et al., 1986; Young and Yamane, 1993; O'Reilly, 1998; McClelland, 2001). Because we do not know the exact dynamics that govern these units, where they would be localized in the brain with respect to each other, and so on, it is virtually impossible to draw any straight and well-defined connection, such as those required to generate clear predictions, between mental units and neural units. Indeed, some studies have challenged the idea of distributed representations and have stood in defense of the so-called *grandmother cells* (e.g., Quiroga et al., 2005; Bowers, 2009). This would point to an easy, one-to-one link function between mental and brain units; but then one needs to consider that (1) most grandmother cell studies have also highlighted massive redundancy in single-cell coding, i.e., there might be many cells coding for, e.g., the word *sofa* (e.g., Waydo et al., 2006); (2) we have no idea on where exactly to expect each relevant cell to be localized in the brain; and (3) widespread imaging techniques are currently far away from recording the activity of single neurons, or small clusters of neurons. Although there are signs that this latter problem might be overcome in a reasonably close future (e.g., Sahin et al., 2009), at least the former two points make clear that even hypothesizing a one-to-one mapping between mind and brain units would hardly be of any help in deriving testable predictions on neural data from (computational) models of cognition. Of course, one-to-one mapping between cognitive and bran units could logically emerge at higher levels of complexity, i.e., between mental processes and cortical areas, rather than between individual representations and single cells. However, experimental data indicate that this is not the case: there seems to be no single brain area that could be held responsible for one single mental operation, and even considering smaller sets of neurons, such as those tracked by cortical stimulation in awake patients, most brain units take part in different cognitive processes (e.g., Roux et al., 2012). These considerations all drive to think that not only existing cognitive models of visual word identification take no stance as to their neural substrates, but also that this would be far from our grasp, given what we currently know. It is important to stress that this is not even close to suggesting that brain data bear no relevance for cognitive theories. What we are saying is, more modestly, that neural effects should not be included into a list of to-be-explained facts because we do not know how *exactly* cognitive and brain units map into each other, and thus we cannot derive exact predictions on brain data on the basis of purely cognitive models.

A final important point raised by Koester (2012) concerns the fact that our target list

“comprises aspects of experimental techniques (masking)” (p. 2, 1st column, 2nd paragraph)

which is questionable because

“Masking does not pertain to the phenomenon in question.” (p. 2, 1st column, 2nd paragraph)

Of course we agree with Koester (2012) that task-related aspects do not belong to the domain of morphology. However, they do make a difference for morphological effects. For example, *corner* is an effective prime for *corn* only when it is presented in a masked form (e.g., Rastle et al., 2000). Or again, *brother*—when compared to *brothel*—makes it easier to identify *broth* in lexical decision (Rastle et al., 2004), but not in a same-different task (Duñabeitia et al., 2011) or in a semantic task (Marelli et al., in press). If a theory refuses to take a position as to how readers carry out these different tasks, what should it account for in these cases? Should it care to explain why *corner* facilitates *corn*, or rather why *corner* does not facilitate *corn*? It is clear that experimental effects only make sense in context, i.e., in specific tasks, because they *always* emerge in specific tasks. This is why we included aspects of experimental techniques in our target list, of course limiting ourselves to those aspects that modulate morphological effects.

In conclusion, we thank Koester (2012) for raising these issues, thus giving us the possibility to clarify our opinion where perhaps we were not clear enough in our original paper. We hope that the notes illustrated in this article will help readers to better understand the sense of our proposal of a target list, and to use this list properly so as to advance our knowledge on how human readers identify printed complex words in a more cooperative and incremental fashion.

## Funding

This research was supported by funding from the Italian Ministry of Education, University, and Research to Davide Crepaldi.
